# Antibodies against tick-borne pathogens in domestic dogs in Norway: *Borrelia burgdorferi* sensu lato, tick-borne encephalitis virus, and *Anaplasma phagocytophilum*

**DOI:** 10.1186/s13028-026-00863-8

**Published:** 2026-03-15

**Authors:** Hanne Kloster, Camilla Stormo, Anita Haug Haaland, Wenche Okstad, Åshild Kristine Andreassen, Snorre Stuen, Vivian Kjelland

**Affiliations:** 1 Faculty of Engineering Science, Department of Natural Sciences, University of Agder, Gimlemoen 25, NO-4630 Kristiansand, Norway; 2 Faculty of Veterinary Medicine, Department of Companion Animal Clinical Sciences, Norwegian University of Life Sciences, Elizabeth Stephansens vei 15, NO-1430 Ås, Norway; 3 Department of Production Animal Clinical Sciences, Section of Small Ruminant Research and Herd Health, Norwegian University of Life Sciences, Svebastadveien 112, NO-4325 Sandnes, Norway; 4 Division for Infection Control and Environmental Health, Department of Virology, Norwegian Institute of Public Health, P. O. Box 4404, Nydalen, NO-0403 Oslo, Norway; 5 Department of Natural Sciences and Environmental Health, University College of Southeast Norway, Gullbringveien 38, NO-3800 Boe, Norway

**Keywords:** Canine health, IgG, Immunoblot, *Ixodes ricinus*, Sentinel species

## Abstract

**Background:**

Ticks are important vectors of zoonotic pathogens, posing a growing threat to animals and humans in Europe. In Norway, the tick species *Ixodes ricinus* is expanding its range, increasing the risk of tick-borne infections. This study aimed to estimate the seroprevalence of IgG antibodies against *Borrelia burgdorferi* sensu lato (s. l.), tick-borne encephalitis virus (TBEV), and *Anaplasma phagocytophilum* in domestic dogs residing in Norway, and to assess their potential role as sentinel species for tick-borne pathogen surveillance at the northernmost limit of *I. ricinus*’ geographical range. Serum samples (*n* = 433) from domestic dogs were collected across 15 counties between 2016 and 2023 and analysed using immunoblot assays.

**Results:**

Overall, 38% of the dogs were seropositive for one or more tick-borne pathogens. The seroprevalence was 20% for *B. burgdorferi* s. l., 19% for TBEV, and 11% for *A. phagocytophilum.* Statistically significant regional differences were observed for the latter two pathogens.

**Conclusions:**

The findings indicated widespread exposure of Norwegian dogs to tick-borne pathogens and support their role as sentinel species for assessing human risk. Continued surveillance and preventive measures are recommended to reduce tick infestation and pathogen transmission to both dogs and humans.

**Supplementary Information:**

The online version contains supplementary material available at 10.1186/s13028-026-00863-8.

## Background

Ticks are globally recognized as important vectors of disease, capable of transmitting a broad spectrum of viral, bacterial, and protozoan pathogens to vertebrate hosts [1]. In Europe, *Ixodes ricinus* serves as the primary vector of tick-borne pathogens affecting both humans and animals [2, 3]. In Norway, this tick species is predominantly distributed along the coastal regions, ranging from Østfold County in the southeast up to Brønnøysund in Nordland County in the north [4–6]. Tick infestation in dogs has been well documented across Europe [3]. Although nymphal ticks are more abundant than adult ticks in the environment [7], studies indicate that the ticks removed from dogs are predominantly adults, most likely because the small size of juvenile tick stages allows them to remain undetected in the dogs’ fur [8]. This indicates that dogs are at a high risk of acquiring tick-borne infections. However, as canine tick-borne infections are not notifiable in Norway, the number of infected or clinically affected dogs remains unknown.

Clinically relevant infections reported in dogs include those caused by viral agents such as tick-borne encephalitis virus (TBEV) and louping ill virus; bacterial pathogens within the *Borreliae*,* Ehrlichiae/Anaplasmae*, and *Rickettsiae* families; and protozoan parasites such as *Babesia* spp., all of which contribute to the overall disease burden in endemic areas [9]. Among the most well-known tick-borne pathogens affecting canines in northern Europe are *Borrelia burgdorferi* sensu lato (s. l.), TBEV, and *Anaplasma phagocytophilum* [3, 10–12].

Lyme borreliosis (LB), caused by *B. burgdorferi* s. l., is the most frequent tick-borne human disease in Europe, with an increasing impact on public health [13]. In Norway, the number of reported cases of systemic LB has doubled over the past decade, from 323 cases in 2014 to 643 cases in 2024 [14]. Although *B. burgdorferi* s. l. infects a variety of wild and domestic animals, the clinical outcomes vary depending on the animal species [3]. In dogs, infections are often mild and self-limiting, and the majority of seropositive individuals remain asymptomatic [12]. When clinical signs do occur, they may include intermittent lameness, joint pain, fever, and lymphadenopathy, and they can appear several months after tick exposure [12, 15, 16].

Tick-borne encephalitis (TBE) is an emerging zoonotic disease in Europe, posing a growing threat to both animal and human health. Although TBEV infection in dogs is primarily subclinical, clinical cases tend to be acute, severe, and frequently fatal [17]. Reported clinical signs include fever, behavioral changes, motor deficits, and multifocal neurological dysfunction [10, 17, 18]. Furthermore, it has been reported that the clinical presentations were consistent with inflammatory central nervous system disorders such as meningitis, meningoencephalitis, and meningomyelitis [10]. These findings underscore the significance of acknowledging the neurological effects of TBE infections in dogs.

Tick-borne fever, caused by *A. phagocytophilum*, is regarded as the most prevalent tick-borne disease in domestic animals in Europe [19], and canine cases are reported worldwide [11]. *A. phagocytophilum* is an intracellular bacterium that primarily infects neutrophil granulocytes [20]. Although infection is common in endemic areas, the majority of dogs exposed to *A. phagocytophilum* appear to remain clinically healthy, as evidenced by widespread seropositivity despite a lack of documented clinical illness [11]. In dogs that develop clinical signs, infection is typically self-limiting and characterized by non-specific symptoms such as lethargy and fever, usually emerging after an incubation period of 1–2 weeks [21]. The most consistent laboratory finding is thrombocytopenia, which is observed in approximately 90% of affected dogs [21]. Hence, tick-borne fever infection may present as an acute febrile illness with low platelet counts [11, 22]. *A. phagocytophilum* evades host immune responses by impairing neutrophil function, which can predispose infected dogs to co-infection with other tick-borne pathogens, potentially complicating diagnosis and exacerbating clinical signs [23, 24].

Dogs are considered suitable hosts for ticks due to their behaviour and physical characteristics, and it has been suggested that dogs may even contribute to sustaining local tick populations, particularly in urban areas where alternative host animals are sparse [8]. Their exploration of nature increases their likelihood of exposure to questing ticks, and frequent movement through vegetation, such as tall grass and underbrush, places them at an ideal height for tick attachment. Additionally, their dense fur can make it difficult to spot attached ticks, providing sufficient time for pathogen transmission from the tick to the dog. Given their risk of tick-borne infections and the following development of detectable antibody responses, dogs can serve as valuable sentinel animals [25, 26]. Because dogs often accompany their owners during outdoor activities, their tick exposure may also mirror that of humans, making them particularly useful for assessing human exposure risk [25, 26]. Hence, monitoring canine exposure may contribute important epidemiological data relevant to veterinary and public health. The present study aimed to estimate the seroprevalence of IgG antibodies against *B. burgdorferi* s. l., TBEV, and *A. phagocytophilum* in domestic dogs residing in Norway, and to assess their potential role as sentinel species for tick-borne pathogen surveillance at the northernmost limit of *I. ricinus*’ geographical range.

## Methods

### Sample collection

**Fig. 1:**
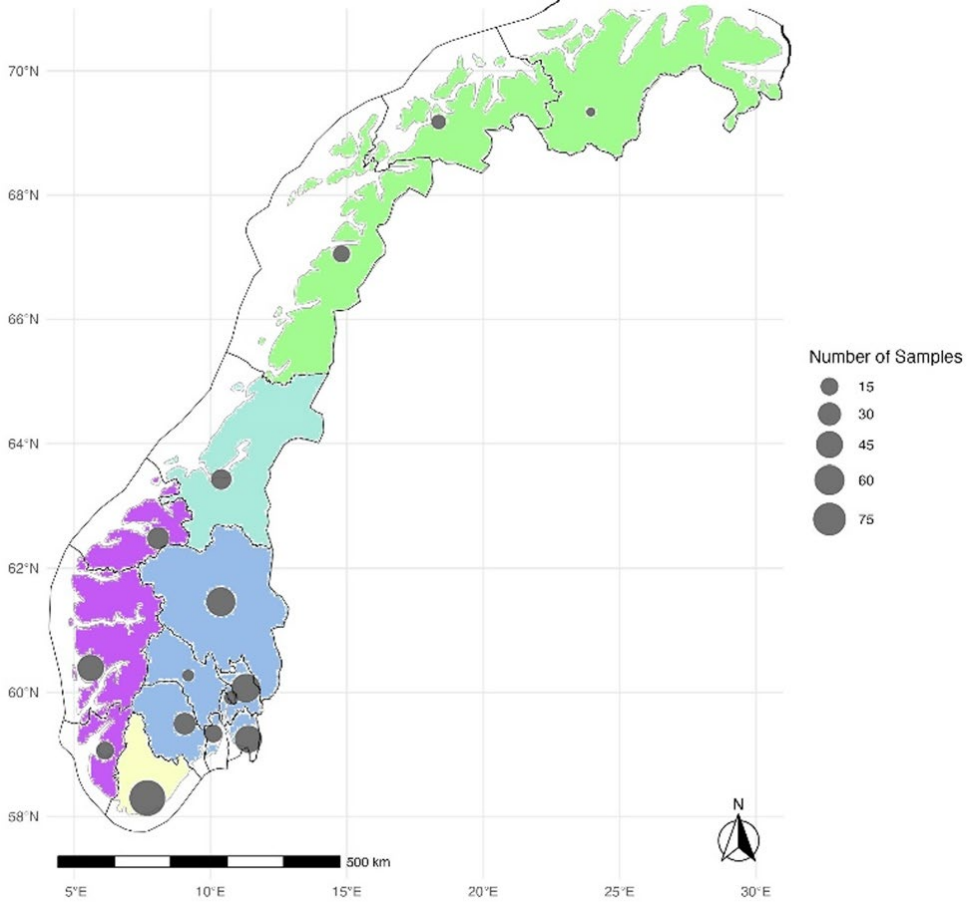
Blood samples were collected from dogs across Norway. The number of included animals at each location is represented by dot size, while the different colours represent the five regions: Eastern (blue), Southern (yellow), Western (purple), Central (turquoise), and Northern Norway (green)

Blood samples from dogs were collected across 15 counties representing all five regions of Norway: Eastern, Southern, Western, Central, and Northern (Fig. 1). Sampling was conducted from June 2016 to October 2023 using a non-systematic recruitment strategy. The study population comprised dogs of various ages, breeds, and both sexes, including neutered individuals. All dogs were already scheduled for veterinary consultations unrelated to tick-borne diseases during the study period, and additional blood samples were collected for research purposes, with oral consent from the dog owners. This was documented by the attending veterinarian in the medical record/journal. In addition, residual canine sera from clinical samples donated by dog owners were also obtained through the Central Laboratory at the Norwegian University of Life Sciences. In all cases, ethical approval for animal experiments was not required, as all dogs were already undergoing routine blood sampling as part of their veterinary visit. The blood samples were collected by veterinarians or veterinary nurses and allowed to clot at room temperature for 30 min before centrifugation at 2000 x g for 15 min. The serum was removed, aliquoted in microtubes, pseudonymized, stored at -20 ˚C, and transported to the University of Agder for further analyses.

### Detection of antibodies in dog sera by immunoblot

Sera were analyzed by the EUROLINE Tick-Borne Profile 1 Dog (IgG) kit (EUROIMMUN Medizinische Labordiagnostika AG, 23560 Lübeck, Germany), according to the manufacturer’s instructions. The immunoblot assay targets IgG-antibodies against *B. burgdorferi* s. l. (VIsE and OspC (p25)), TBEV (gpE), and *(A) phagocytophilum* (MSP-2). According to the manufacturer, due to the high quality of the antigen substrate, no cross-reactivity between *(B) burgdorferi* s. l. and other species are expected. However, cross-reactions between *(A) phagocytophilum* and *Anaplasma platys*, *Ehrlichia* spp., and *Rickettsia* spp. have been reported. Furthermore, cross-reactivity between TBEV and other flaviviruses cannot be excluded; however, the use of recombinant antigens significantly reduces the probability of such occurrences. According to the manufacturer, the sensitivity/specificity of the test were as follows: 100%/100% for antibodies against *(B) burgdorferi* s. l., 93%/91% for TBEV and 100%/91% for *A. phagocytophilum*. Results were automatically evaluated and classified as positive, negative, or borderline by the EUROLineScan program. Borderline results were interpreted as negative.

### Calculation of human lyme borreliosis and tick-borne encephalitis incidence

The number of registered cases of human Lyme borreliosis and tick-borne encephalitis (TBE) was obtained from the Norwegian Surveillance System for Communicable Diseases (MSIS) [14, 27]. The annual number of reported Lyme borreliosis cases in each region (county) was extracted for 2024, and incidence was calculated as the number of cases per 100,000 inhabitants per year. Population data for the corresponding year were retrieved from Statistics Norway [28].

### Statistics

Statistical analyses were performed with STATA/SE 17.0 (STATA Corp LLC., College Station, TX, USA, 2021). The Pearson Chi-square test was used to assess differences in seroprevalence across regions. Logistic regression analyses were conducted to assess whether observed regional differences in seroprevalence persisted after adjusting for potential confounders such as age, sex, and breed.

The correlation between canine *Borrelia* seroprevalence and reported human Lyme borreliosis incidence, as well as between canine TBEV seroprevalence and reported human TBE cases, respectively, across regions was assessed using linear regression analysis. The Clopper-Pearson exact method was used to calculate proportions of 95% confidence intervals (CI). A significance level of *P* < 0.05 was considered statistically significant.

### Spatial visualization

The distribution of collected samples was mapped by R (version 2023.09.1 + 494) [29] with the ggplot2 (version 3.5.1) [30] and sf (version 1.0–20) [31] packages. Municipality boundaries were obtained from the rnaturalearth package, and sample locations were aggregated based on unique spatial coordinates.

## Results

### Demographic characteristics

A total of 433 dogs from across Norway were included in the study. The age of the dogs ranged from six weeks to 16 years; 26 puppies (0–1 year), 27 young dogs (1–2 years), 156 adults (2–8 years), 164 seniors (> 8 years), and 60 unreported. Further, the distribution between sexes was 184 females, 164 males, and 85 unreported. The dogs represented a diverse range of breeds, with 106 distinct breeds identified (Additional file 1). The most common breeds, aside from mixed breeds (*n* = 82), were Border Collie (*n* = 17), followed by English Setter (*n* = 12), Golden Retriever (*n* = 11), Gordon Setter (*n* = 10), and Irish Wolfhound (*n* = 10).

### Seroprevalence of tick-borne pathogens in dogs

In total, 38% (165/433) of the dogs were seropositive for IgG antibodies against one or more tick-borne pathogens (*B. burgdorferi* s. l., TBEV, and/or *(A) phagocytophilum)*, with regional seroprevalences ranging from 22% (6/27) in Northern Norway to 52% (48/93) in Southern Norway. Among the seropositive dogs, 73% showed evidence of exposure to a single pathogen, while 22% were seropositive for two pathogens, and 4% for three pathogens (Additional file 2). The seroprevalence of individual pathogens was 20% (CI: 16–24) for *(B) burgdorferi* s. l., 19% (CI: 16–23) for TBEV, and 11% (CI: 8–14) for *A. phagocytophilum*. A detailed overview of seroprevalence across all 15 counties is provided in Table 1.

**Table 1:** Seroprevalence of IgG antibodies against tick-borne pathogens in dogs residing in Norway

Regions (*n*)	County (*n*)	Total Bbsl % (*n*) (CI)	Total TBEV % (*n*) (CI)	Total Ap % (*n*) (CI)
Eastern Norway (208)		18 (38) (13–24)	20 (41) (14–26)	2 (5) (1–6)
	Innlandet (54)	7 (4)	17 (9)	0
	Akershus (53)	19 (10)	19 (10)	2 (1)
	Buskerud (6)	50 (3)	17 (1)	17 (1)
	Oslo (8)	13 (1)	38 (3)	0
	Østfold (46)	9 (4)	20 (9)	2 (1)
	Vestfold (15)	27 (4)	13 (2)	0
	Telemark (26)	46 (12)	27 (7)	8 (2)
Southern Norway (93)		25 (23) (16–35)	28 (26) (19–38)	22 (20) (14–31)
	Agder (93)	25 (23)	28 (26)	22 (20)
Western Norway (84)		20 (17) (12–30)	11 (9) (5–19)	23 (19) (14–30)
	Rogaland (15)	20 (3)	13 (2)	33 (5)
	Vestland (43)	16 (7)	12 (5)	23 (10)
	Møre & Romsdal (26)	27 (7)	8 (2)	15 (4)
Central Norway (21)		19 (4) (5–42)	24 (5) (8–47)	14 (3) (3–36)
	Trøndelag (21)	19 (4)	24 (5)	14 (3)
Northern Norway (27)		11 (3) (2–29)	11 (3) (2–29)	0 (0–13)
	Nordland (13)	15 (2)	0	0
	Troms (9)	11 (1)	22 (2)	0
	Finnmark (5)	0	20 (1)	0
Total (*n* = 433)		20 (85) (16–24)	19 (84) (16–23)	11 (47) (8–14)

Regional seroprevalence against *Borrelia burgdorferi* sensu lato (Bbsl), tick-borne encephalitis virus (TBEV), and *Anaplasma phagocytophilum* (Ap) in dogs residing in Norway

**Fig. 2:**
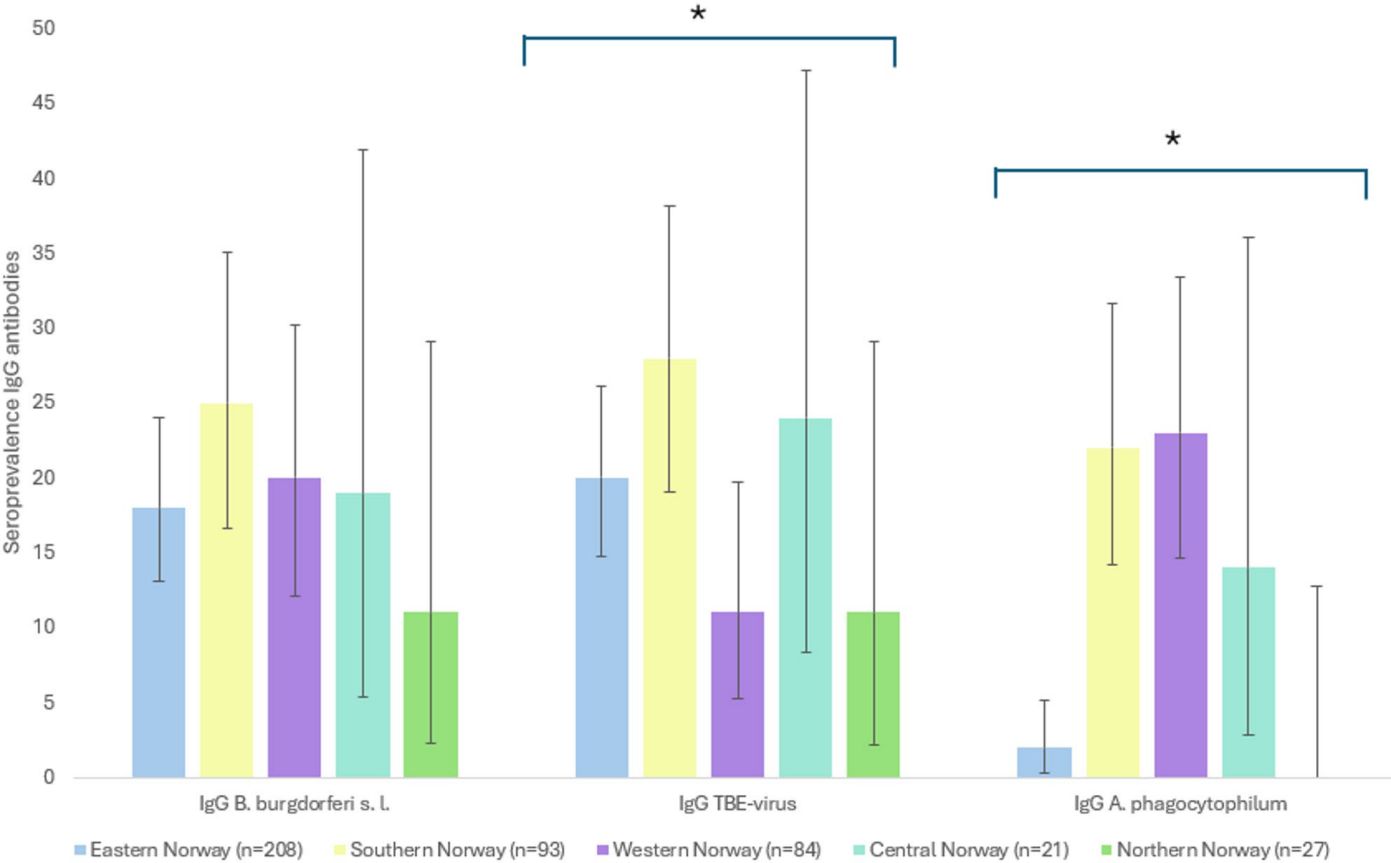
Seroprevalence of IgG antibodies against *B. burgdorferi* s. l., TBEV, and *A. phagocytophilum* in dogs residing in regions across Norway. Asterisks (*) denote statistically significant overall variation across regions. Error bars represent 95% confidence intervals

When comparing across regions, no statistically significant differences in the seroprevalence of *B. burgdorferi* s. l. were detected (*P* = 0.180), although the rates varied from 11% (CI: 2–29) in Northern Norway, to 18% (CI: 13–24) in Eastern Norway, 19% (CI: 5–42) in Central Norway, 20% (CI 12–30) in Western Norway, and 25% (CI: 16–35) in Southern Norway (Fig. 2). In contrast, statistically significant regional differences in seroprevalence were observed for TBEV (*P* = 0.043). The highest seroprevalence was found in Southern Norway (28%, CI: 19–38), followed by Central (24%, CI: 8–47) and Eastern Norway (20%, CI: 14–26), whereas the lowest was seen in Northern (11%, CI: 2–29) and Western Norway (11%, CI: 5–19). Similarly, a highly significant regional difference was detected for *A. phagocytophilum* (*P* = 0.0001), with the highest seroprevalence observed in Western (23%, CI: 14–30), Southern (22%, Cl: 14–31), and Central Norway (14%, CI: 3–36), compared to a markedly lower seroprevalence in Eastern Norway (2%, CI: 1–6) and no positive samples detected in Northern Norway (0%, CI: 0–13). These statistically significant regional differences for TBEV and *A. phagocytophilum* persisted after adjusting for age, sex, and breed, indicating robust geographical variation in exposure.

Seroprevalence by age, sex, and breed is presented in Table 2. A significant difference was observed between age groups when analyzing for IgG antibodies against *(A) phagocytophilum* (*P* = 0.003), with the highest seroprevalence detected among senior dogs (17%, 27/164), followed by adults (6%, 10/156), puppies (4%, 1/26), and young dogs (0%, 0/27). In contrast, no significant differences were detected between age groups for antibodies against *(B) burgdorferi* s. l. and TBEV. For the most represented breeds and between sexes, there were no significant differences for any of the tick-borne pathogens. However, it should be noted that none of the 17 Border Collie samples tested positive for IgG antibodies against *B. burgdorferi* s. l.

**Table 2:** Seroprevalence against tick-borne pathogens by age, sex, and breed in the sampled dog population

Variable	Category	Number of dogs (*n* = 433)	Bbsl	TBEV	Ap
			% (*n*)	% (*n*)	% (*n*)
Age	Puppy (0–1 year)	26	23 (6)	12 (3)	4 (1)
	Young (1–2 years)	27	26 (7)	15 (4)	0 (0)
	Adult (2–8 years)	156	21 (32)	22 (32)	6 (10)
	Senior (> 8)	164	18 (30)	19 (31)	17 (27)
	n/a^1^	60	17 (10)	23 (14)	15 (9)
Breed	Border Collie	17	0 (0)	24 (4)	6 (1)
	English Setter	12	17 (2)	33 (4)	8 (1)
	Golden Retriever	11	18 (2)	27 (3)	18 (2)
	Gordon Setter	10	30 (3)	50 (5)	10 (1)
	Irish Wolfhound	10	30 (3)	0 (0)	0 (0)
	Mixed breed	82	23 (19)	23 (19)	11 (9)
	Other^2^, n/a^1^	291	19 (56)	17 (49)	11 (33)
Sex	Female	184	15 (28)	21 (39)	9 (16)
	Male	164	20 (33)	17 (28)	11 (18)
	n/a^1^	85	26 (22)	19 (16)	13 (11)

Seroprevalence against *Borrelia burgdorferi* sensu lato (Bbsl), tick-borne encephalitis virus (TBEV), and *Anaplasma phagocytophilum* (Ap) by age, sex, and breed in the sampled dog population

^1^n/a: not available

^2^Other: Breeds with fewer than 10 dogs

### Correlation between canine seroprevalence and human lyme borreliosis incidence and tick-borne encephalitis

Regions with high canine *B. burgdorferi* s. l. seroprevalence corresponds to the reported high human Lyme borreliosis incidence (R²=0.739; Table 3). This suggests that approximately 74% of the variation in human incidence could be explained by regional differences in canine seroprevalence. In contrast, no correlation was observed between canine seroprevalence of TBEV and human TBE incidence. All TBE infections reported were restricted to Southern and Eastern Norway.

**Table 3:** Canine seroprevalence and human Lyme borreliosis and tick-borne encephalitis incidence in Norway

Region	Seroprevalence in dogs (2016–2023) (%)		Human incidence (2024) (per 100,000 inhabitants)		
	Bbsl	TBEV	LB	TBE	
Eastern Norway	18.2	19.7	6.5		1.0
Southern Norway	24.7	27.9	27.2		5.9
Western Norway	20.2	10.7	23.3		0
Central Norway	19.0	23.8	7.5		0
Northern Norway	11.1	11.1	1.0		0

Canine seroprevalence of *Borrelia burgdorferi* sensu lato (Bbsl) and tick-borne encephalitis virus (TBEV), in addition to human incidence (cases per 100,000 inhabitants) of Lyme borreliosis (LB) and tick-borne encephalitis (TBE) in the five regions of Norway

## Discussion

In total, 38% of the dogs in the present study had antibodies against one or more of the pathogens investigated. IgG antibodies against tick-borne pathogens were detected in dogs from all regions in Norway, including northern regions where few established populations of *I. ricinus* have been confirmed [32]. *I. ricinus* is the predominant tick species in Norway [5, 6, 32] and is therefore the most likely source of pathogen exposure. However, exposure to other tick species cannot be excluded, nor can the possibility that some dogs acquired infection outside their county of residence, as travel history was not available for the included dogs. Furthermore, it has been demonstrated that infected dams may transfer maternal antibodies against *B. burgdorferi* s. l. to their pups [33], and vertical transmission of TBEV is suspected [34]. Hence, we cannot exclude the possibility that IgG-positive puppies acquired maternally derived antibodies or were infected vertically, rather than through tick exposure.

### Seroprevalence of IgG antibodies against tick-borne pathogens

The overall seroprevalence of IgG antibodies against *B. burgdorferi* s. l. was 20%, ranging from 11 to 25%, depending on geographic region. Although no statistically significant differences between regions were seen, the highest seroprevalence was observed in Southern Norway, consistent with high tick population densities as well as previous reports of high prevalence of *B. burgdorferi* s. l. in questing ticks in this region [7]. This aligns with findings reported nearly three decades earlier from the same region, where the seroprevalence of *B. burgdorferi* s. l. in dogs was 14% and 27%, respectively [35, 36]. In contrast, our data indicate a different trend in Northern Norway: A previous report published in 2012 found a canine seroprevalence of only 1.7% (2/120) in the northern region [37], whereas the present study found a higher seroprevalence of 11% (3/27). Although this may suggest a rise in exposure in northern regions, the small sample size in our study warrants cautious interpretation. Notably, the canine seroprevalence of *B. burgdorferi* s. l. in Nordic countries seems highly variable; 2.9% in Finland [38], 3.9% in Sweden [39], 16.1% in Denmark [40], and 20% in Norway. Also, a recent multicentre study reported an overall canine seroprevalence of 2.4% in Europe, but also here local results ranged widely, from 0 to 13.3% [41]. The seroprevalence is likely influenced by several factors, such as regional variation of tick abundance, environmental conditions affecting tick activity, intensity of local pathogen transmission, and levels of tick exposure in the sampled canine population. In addition, differences in test performance may influence seroprevalence estimates. When seroprevalence is interpreted in the context of the immunoassay’s reported sensitivity and specificity (both 100%), the positive results in our study likely represent true previous exposure to *B. burgdorferi* s. l.

In the present study, the overall seroprevalence of IgG antibodies against TBEV was 19%, and seropositive dogs were seen in all regions. Regional seroprevalence ranged from 11% in Northern Norway to 28% in Southern Norway. The prevalence of TBEV in questing ticks collected in Norway is also highly variable, depending on region, ranging from 0 to 20.6% [6, 42]. Although several factors may influence the risk of infection, the varying endemicity of TBEV in different regions may partly explain the marked regional differences in seroprevalence observed in this study. Widely varying seroprevalence of TBEV in dogs is also seen across Europe, ranging from 0 to 40% depending on geographic location, endemicity, and study design [3, 43–46]. Previously, a study conducted in southern Norway found that 16% of dogs were seropositive [47]. The higher seroprevalence in the present study may partly be explained by differences in assay methodology, but could also reflect a true increase in tick abundance or infected ticks. Supporting this notion, there has been a general increase in reported human TBE cases in Norway over the past two decades [14]. The current study included limited sample size from Central and Northern Norway, warranting cautious interpretation of the results. Furthermore, cross-reaction with other flaviviruses cannot be excluded [48], and positive TBEV results should be interpreted with caution. Nevertheless, our findings indicate that dogs are at risk of acquiring tick-borne viral infection, and TBE should be considered among the differential diagnoses in dogs with neurological symptoms.

The overall seroprevalence of IgG antibodies against *(A) phagocytophilum* in dogs in the present study was 11%, with regional variations ranging from 0 to 23%. These findings fall within previously reported seroprevalence rates across Europe, which range from 0 to 43.3% [21, 38, 41, 49–52]. As for *(B) burgdorferi* s. l. and TBEV, the wide variability may be caused by several factors. In Norway, the prevalence of *A. phagocytophilum* in questing ticks has also been shown to vary greatly between sites, from 0 to 17.5% [53–57], and may partly explain the regional differences in canine seroprevalence. Again, due to limited sample size, the estimated seroprevalences in Northern and Central Norway should be interpreted with caution. Also, cross-reaction cannot be excluded, and *Neoerlichia mikurensis* is previously suggested to be the most likely agent cross-reacting with *A. phagocytophilum* in Norway [48].

Co-infection with tick-borne pathogens is assumed to increase disease severity; however, the clinical consequences of such co-infections remain poorly understood [58]. In the present study, antibodies against two or three pathogens were detected in 10% (44/433) of the included dogs, but due to the persistence of antibodies after prior pathogen exposure [16, 44, 59], we cannot determine whether these dogs were concurrently infected or whether the observed serological patterns reflect sequential exposures, nor can we assess any associated impact on the dogs’ health.

### Breed-related risk factors

Breed-related risk factors such as activity level, primary use (e.g. hunting dog vs. companion dog), outdoor environment, coat characteristics, grooming practices, and the use of tick ectoparasiticides may influence the risk of tick bites and pathogen transmission. In addition to behavioural and environmental factors, differences in host-derived volatile organic compounds may influence host-seeking behaviour in ticks [60]. Recent evidence suggests that such volatiles can affect tick attraction and potentially vector competence, although breed-specific differences in dogs remain unexplored. In Norway, there are approximately 590,000 dogs, present in about 18% of all households [61]. In our study, Border Collie, Golden Retriever, and Setter breeds were most represented. These breeds rank among the most popular in Norway, based on annual registrations reported by the Norwegian Kennel Club and a national data registry [61, 62]. In contrast, the Irish Wolfhound is rare, with fewer than 200 members in the breed-specific Norwegian Kennel Club [62]. Border Collie, Setter breeds, and often Golden Retrievers are frequently used in outdoor activities such as herding, hunting, or companionship, making them prone to tick exposure. The long and dense coat of some breeds, such as the Golden Retriever, may also facilitate prolonged tick attachment. The Border Collies were clearly exposed to ticks as they showed seroprevalence of antibodies against TBEV and *(A) phagocytophilum*, yet none of the Border Collies was positive for antibodies against *(B) burgdorferi* s. l. Half of the Gordon Setters were seropositive for antibodies against TBEV, and the Irish Wolfhounds were negative for antibodies against TBEV and *(A) phagocytophilum* but had a relatively high seroprevalence of *(B) burgdorferi* s. l. These findings could not be linked to the geographical distribution of the dogs, and none of the breed-related risk factors commonly referred to seems plausible. The sample sizes in these cohorts are very small, and the findings could be due to chance. However, previous studies have also reported breed predispositions [63, 64]. Several studies have confirmed a particularly high seroprevalence of *B. burgdorferi* s. l. in Bernese Mountain dogs, but not against other tick-borne diseases in the same region [64]. There were seven Bernese Mountain dogs in this study, of which one tested positive for *B. burgdorferi* s. l. Further studies with larger breed-specific cohorts are warranted to explore potential breed-related risk factors.

### Dogs as sentinel animals for human lyme borreliosis and tick-borne encephalitis

Seropositivity does not necessarily indicate active infection but may reflect prior or subclinical exposure. While it is important to consider the clinical relevance of these infections for canine health, dogs may also serve as valuable sentinels for the surveillance of tick-borne pathogens. In the present study, we observed a positive association between canine *Borrelia* seroprevalence and human incidence of LB [14, 28]. These findings are consistent with previous work demonstrating a quantitative relationship between *B. burgdorferi* exposure in domestic dogs and human LB incidence, supporting the potential use of canine seroprevalence as a proxy for human risk of LB [65]. In contrast, the canine seroprevalence of TBEV did not correlate with the incidence of human TBE cases; however, the dogs still serve as valuable sentinels for vector and pathogen distribution surveys. Interestingly, there have been no reported human TBE cases from Western Norway [14, 27], despite 11% of the dogs from this region testing positive for antibodies against TBEV. These findings are supported by evidence of TBEV in questing ticks (0 to 20%) [42] and a seroprevalence of 10% in horses from the same region [48]. The reason for the lack of human cases in this region is so far unknown, but could be due to multiple intrinsic and extrinsic factors of host and vectors or variants of the pathogens causing asymptomatic infections [42, 66]. This geographic discrepancy between canine seroprevalence and lack of reported human cases highlights the potential value of dogs as sentinel species in surveillance efforts, as canine serology may serve as an early indicator of TBEV circulation in areas where clinical cases have yet to be detected.

### Limitations of this study

The non-systematic sampling approach may have affected the representativeness of the results. For instance, clinics or dog owners with a particular interest in tick-borne diseases may have been more likely to participate. To mitigate this potential bias, we aimed to include samples from areas outside *I. ricinus*’ geographical range where interest in ticks and tick-borne infections tends to be lower, and participation was less likely to be driven by heightened concern about ticks. A larger and more systematically sampled study is needed to further investigate regional differences and to evaluate potential changes in seroprevalence between years and over time. Nevertheless, the dataset represents the most geographically comprehensive survey of canine exposure to tick-borne pathogens conducted in Norway to date, and the detection of pathogen exposure outside the established range of *I. ricinus* underscores the importance of broad-scale surveillance efforts.

## Conclusions

This study demonstrates that domestic dogs throughout Norway are exposed to ticks and tick-borne infections, with 38% of the sampled population seropositive for one or more of the pathogens *B. burgdorferi* s. l., TBEV, and *A. phagocytophilum.* IgG antibodies were detected across all age groups, both sexes, and a broad range of breeds. Given their close contact with humans and their high likelihood of tick exposure, dogs represent a valuable sentinel species for monitoring the distribution and circulation of tick-borne pathogens, and for human LB. Continued surveillance, increased public awareness, and the implementation of preventive measures, such as the use of tick ectoparasiticides in consultation with a veterinarian, along with regular tick checks, are recommended to reduce the risk of pathogen transmission and exposure for both dogs and their owner.

## Supplementary Information

Below is the link to the electronic supplementary material.

Supplementary Material 1.

Supplementary Material 2.

## Data Availability

The datasets used and/or analyses during the current study are available from the corresponding author on reasonable request.

## Declarations

### Prior publication

Data have not been published previously.
