# Supplementary material for: Antibodies against tick-borne pathogens in domestic dogs in Norway: Borrelia burgdorferi sensu lato, tick-borne encephalitis virus, and Anaplasma phagocytophilum
Source: Acta Vet Scand. 2026 Mar 15;68:22. doi: 10.1186/s13028-026-00863-8 (PMC13104503; doi:10.1186/s13028-026-00863-8)
Supplement: Supplementary file 1 — Supplementary Material 1. [file 13028_2026_863_MOESM1_ESM.docx]

**Additional file 1:** Overview of breeds included in the study

| **Breed** | **Number** |
| --- | --- |
| Alaskan Husky | 3 |
| American Cocker Spaniel | 2 |
| Australian Cattle Dog | 1 |
| Australian Cobber dog | 1 |
| Australian Shepherd | 1 |
| Australian Terrier | 1 |
| Bavarian Mountain Hound | 1 |
| Beagle | 1 |
| Belgian Tervuen | 1 |
| Bernese Mountain Dog | 6 |
| Bichon frisè | 6 |
| Bichon Havanese | 1 |
| Border Collie | 17 |
| Bosnian Coarse-haired Hound | 1 |
| Boston Terrier | 1 |
| Boxer | 2 |
| Braque de I'Ariege | 1 |
| Brittany Spaniel | 2 |
| Bull Terrier | 1 |
| Cairn Terrier | 5 |
| Catalan Sheepdog | 1 |
| Cavalier King Charles Spaniel | 5 |
| Chespeake Bay Retriever | 1 |
| Chihuahua | 2 |
| Dachshund (all types) | 6 |
| Dalmatian | 1 |
| Danish-Swedish Farmdog | 5 |
| Dobermann | 2 |
| Dogue de Bordeaux | 1 |
| Dorset Old Tyme Bulldog | 1 |
| Drever | 2 |
| Dunker | 1 |
| English Cocker Spaniel | 2 |
| English Setter | 12 |
| English Springer Spaniel | 5 |
| Eurasier | 4 |
| Field Spaniel | 1 |
| Finnish Hound | 4 |
| Finnish Lapphund | 5 |
| Finnish Spitz | 1 |
| Flat-coated Retriever | 7 |
| French Bulldog | 3 |
| German Hunt Terrier | 1 |
| German Shepherd Dog | 8 |
| German Spitz Klein | 2 |
| German Spitz Mittel | 1 |
| Giant Schnauzer (black) | 1 |
| Golden Retriever | 11 |
| Gordon Setter | 10 |
| Greyhound | 1 |
| Halden Hound | 1 |
| Hungarian Vizsla | 1 |
| Irish Setter | 2 |
| Irish Water Spaniel | 1 |
| Irish Wolfhound | 10 |
| Italian Greyhound | 1 |
| Jack Russell Terrier | 3 |
| Japanese Spitz | 3 |
| Jämthund | 5 |
| Keeshond | 1 |
| Kerry Blue Terrier | 4 |
| Kuvasz | 1 |
| Labrador Retriever | 5 |
| Lagotto Romagnolo | 2 |
| Lhasa apso | 1 |
| Maremma Sheepdog | 1 |
| Miniature Poodle | 6 |
| Miniature Schnauzer (salt & pepper) | 1 |
| Mixed breed | 82 |
| Newfoundland | 1 |
| Norwegian Buhund | 1 |
| Norwegian Elkhound Black | 1 |
| Norwegian Elkhound Grey | 6 |
| Petit Basset Griffon Vendèen | 2 |
| Phalene | 1 |
| Pointer | 3 |
| Pomeranian | 5 |
| Poodle | 2 |
| Portuguese Podengo Pequeno | 2 |
| Portuguese Water Dog | 1 |
| Pyrenean Mountain Dog | 1 |
| Rottweiler | 1 |
| Saint Germain Pointer | 1 |
| Samoyed | 1 |
| Schapendoes | 1 |
| Shetland Sheepdog | 4 |
| Shiba Inu | 1 |
| Shih Tzu | 1 |
| Siberian Husky | 3 |
| Silky Terrier | 2 |
| Scottish Deerhound | 2 |
| Small Musterlander | 2 |
| Smålandsstövare | 1 |
| Springer spaniel | 1 |
| Staffordshire Bull Terrier | 6 |
| Standard Poodle | 5 |
| Standard Schnauzer | 1 |
| Tibetan Spaniel | 1 |
| Tibetan Terrier | 2 |
| Toy Poodle | 3 |
| Vorstehhund | 2 |
| West Highland White Terrier | 3 |
| West Siberian Laika | 1 |
| Whippet | 1 |
| White Swiss Shepherd | 2 |
| Yorkshire Terrier | 4 |
| n/a | 62 |
| Total | 433 |
