# Supplementary material for: Antibodies against tick-borne pathogens in domestic dogs in Norway: Borrelia burgdorferi sensu lato, tick-borne encephalitis virus, and Anaplasma phagocytophilum
Source: Acta Vet Scand. 2026 Mar 15;68:22. doi: 10.1186/s13028-026-00863-8 (PMC13104503; doi:10.1186/s13028-026-00863-8)
Supplement: Supplementary file 2 — Supplementary Material 2. [file 13028_2026_863_MOESM2_ESM.docx]

**Additional file 2:** Seropositivity combinations for tick-borne pathogens in dogs across Norway.

|  |  | **Single** |  |  | **Double** |  | **Triple** |  |
| --- | --- | --- | --- | --- | --- | --- | --- | --- |
|  | ***Bb*sl** | **TBEV** | ***Ap*** | **Bbsl + TBEV** | **Bbsl + Ap** | **Ap + TBEV** | **Bbsl + Ap + TBEV** | **Total positive** |
|  | **% (n)** | **% (n)** | **% (n)** | **% (n)** | **% (n)** | **% (n)** | **% (n)** | **% (n)** |
| **Eastern Norway (n=208)** | 12 (24) | 14 (29) | 1 (2) | 5 (11) | 1 (2) | 0 | 1 (1) | 33 (69) |
| **Southern Norway (n=93)** | 12 (11) | 14 (13) | 7 (6) | 4 (4) | 5 (5) | 7(6) | 3 (3) | 52 (48) |
| **Western Norway (n=84)** | 10 (8) | 5 (4) | 14 (12) | 2 (2) | 5 (4) | 0 | 4 (3) | 39 (33) |
| **Central Norway (n=21)** | 10 (2) | 14 (3) | 5 (1) | 5 (1) | 5 (1) | 5 (1) | 0 | 43 (9) |
| **Northern Norway (n=27)** | 11 (3) | 11 (3) | 0 | 0 | 0 | 0 | 0 | 22 (6) |
| **Total (N=433)** | 11 (48) | 12 (52) | 5 (21) | 4 (18) | 3 (12) | 2 (7) | 2 (7) | 38 (165) |

Frequency of single, dual, and triple IgG seropositivity against the tick-borne pathogens *Borrelia burgdorferi* sensu lato (Bbsl), tick-borne encephalitis virus (TBEV), and *Anaplasma phagocytophilum* (Ap) in dogs across Norwegian regions.
